# The late radiotherapy normal tissue injury phenotypes of telangiectasia, fibrosis and atrophy in breast cancer patients have distinct genotype-dependent causes

**DOI:** 10.1038/sj.bjc.6603637

**Published:** 2007-02-27

**Authors:** G Giotopoulos, R P Symonds, K Foweraker, M Griffin, I Peat, A Osman, M Plumb

**Affiliations:** 1 Department of Genetics, University of Leicester, Leicester LE1 7RH, UK; 2 Department of Cancer Studies and Molecular Medicine, University of Leicester, Level 2, Osborne Building, Leicester Royal Infirmary, Leicester LE1 5WW, UK; 3 Department of Oncology, Nottingham University Hospitals NHS Trust, CITY Hospital Campus, ICT Services, Hucknall Road, Nottingham, UK

**Keywords:** breast cancer, radiation injury, TGF*β*1, XRCC1, fibrosis, telangiectasia

## Abstract

The relationship between late normal tissue radiation injury phenotypes in 167 breast cancer patients treated with radiotherapy and: (i) radiotherapy dose (boost); (ii) an early acute radiation reaction and (iii) genetic background was examined. Patients were genotyped at single nucleotide polymorphisms (SNPs) in eight candidate genes. An early acute reaction to radiation and/or the inheritance of the transforming growth factor-*β*1 (*TGFβ1* −509T) SNP contributed to the risk of fibrosis. In contrast, an additional 15 Gy electron boost and/or the inheritance of X-ray repair cross-complementing 1 (*XRCC1*) (R399Q) SNP contributed to the risk of telangiectasia. Although fibrosis, telangiectasia and atrophy, all contribute to late radiation injury, the data suggest that they have distinct underlying genetic and radiobiological causes. Fibrosis risk is associated with an inflammatory response (an acute reaction and/or TGF*β*1), whereas telangiectasia is associated with vascular endothelial cell damage (boost and/or XRCC1). Atrophy is associated with an acute response, but the genetic predisposing factors that determine the risk of an acute response or atrophy have yet to be identified. A combined analysis of two UK breast cancer patient studies shows that 8% of patients are homozygous (TT) for the *TGFβ1* (C-509T) variant allele and have a 15-fold increased risk of fibrosis following radiotherapy (95% confidence interval: 3.76–60.3; *P*=0.000003) compared with (CC) homozygotes.

Radiotherapy is associated with a wide spectrum of normal-tissue reactions, and as the life expectancy of cancer patients increases, normal tissue effects are increasingly of clinical importance. Tissue toxicity may range from asymptomatic changes in tissue structure and function, to severe cosmetic disfigurement and life-altering changes in organ function (Bentzen *et al*, 2003). The effects can be divided into *early/acute* reactions, which occur within 90 days of treatment, and *late* reactions that occur more than 90 days after treatment and can persist for life (Van der Kogel, 1993).

Early reactions principally affect high turn-over tissues, such as skin, the gastrointestinal tract and bone marrow where the onset and severity of the reactions reflect the balance between the rate of stem/progenitor-cell killing and the rate of regeneration of surviving cells (Van der Kogel, 1993). Severe acute reactions are rare and have been associated with syndromes such as Ataxia Telangiectasia and Nijmgen Breakage syndrome that have inherited highly penetrant and mutant alleles encoding DNA repair enzymes, although low penetrance alleles in the general population have also been implicated in cellular radiosensitivity (Hu *et al*, 2001; Moullan *et al*, 2003; Chang-Claude *et al*, 2005; Fernet and Hall, 2005).

Late effects typically, but not exclusively, manifest in low turn-over tissues such as lung, heart, liver and the nervous system that are comparatively depleted of stem and progenitor cells. Common late manifestations include fibrosis, telangiectasia and atrophy, and a particular tissue or organ may show signs of one or more of these reactions at different times following the radiotherapy (O’Sullivan and Levin, 2003). A significant number of patients (∼5%) show late normal tissue damage, so there are presumably genetic component(s) that contribute(s) to the risk and severity of the late tissue radiation effects in the general population.

Transforming growth factor-*β*1 (TGF*β*1) is the major cytokine responsible for the regulation of fibroblast proliferation and differentiation (Randall and Coggle, 1995; Gauldie *et al*, 2003; O’Sullivan and Levin, 2003). Differentiated fibroblasts synthesise the collagens and proteoglycans in the extracellular matrix, and it has been suggested that an increase in these fibroblasts may cause the fibrotic phenotype (Mauch and Kreig, 1990; Martin *et al*, 2000; O’Sullivan and Levin, 2003). Radiation induces long-term TGF*β*1 overexpression probably owing in part to oxidative stress and an inflammatory response (Randall and Coggle, 1995; Anscher *et al*, 1997; Martin *et al*, 1997, 2000; O’Sullivan and Levin, 2003), and a causal relationship between increased TGF*β* production and the development of tissue fibrosis has been reported in animal models (Zugmaier *et al*, 1991; Terrell *et al*, 1993; Sanderson *et al*, 1995; Clouthier *et al*, 1997). Most significantly, elevated serum TGF*β*1 levels were correlated with an increased risk of fibrosis in breast and lung cancer patients (Awad *et al*, 1988; Anscher *et al*, 1997; Grainger *et al*, 1999; Li *et al*, 1999) and a comparison of the genotypes of unaffected and affected patients has been genetically associated with functional polymorphisms in the *TGFβ1* gene (Awad *et al*, 1988; El-Gamel *et al*, 1999; Quarmby *et al*, 2003; Andreassen *et al*, 2005).

Whether the late tissue effects such as fibrosis, atrophy and telangiectasia are biologically or radiobiologically related is controversial. The observation that normal tissue damage can involve one or combinations of these phenotypes suggests that their underlying causes may be different. The power of genetic studies seeking to associate a genotype with a phenotype is absolutely dependent on the precise definition of the phenotype. Several scales have been developed, which allow recording of such injury uniformly and reproducibly, and include the Radiation Therapy Oncology Group (RTOG) scales, which are relatively simple to score as each site treated with radiotherapy scores 0–5 according to the degree of injury incurred with an extreme score of 5 indicating patient death (Cox *et al*, 1995).

The Late Effects of Normal Tissue-Subjective Objective Management Analytical (LENT-SOMA) scale allows comparison between individuals of the clinical perception of injury, the patient's perception of the injury, the medical management required and the underlying pathological process (Pavy *et al*, 1995). A score of 0–4 is given to each of the LENT-SOMA tests, and the sum of the individual scores for each patient a measure of overall tissue damage. However, the *Objective* analyses in LENT-SOMA include separate quantitative measures of fibrosis, atrophy and telangiectasia, so LENT-SOMA provides a more detailed and specific description of the nature (phenotype) and severity of the injury/ies than the RTOG scale.

In this report, we have carried out a genetic association study on breast cancer patients at least 4 years after radiotherapy treatment who were scored for normal tissue damage using both RTOG and SOMA scales. The patients were also scored for an early acute reaction to radiotherapy within 90 days of treatment, and whether or not they received a 15 Gy electron boost. Patients were genotyped for functional single nucleotide polymorphisms (SNPs) in eight candidate genes, and thus enabled the analysis of the relative contribution of radiotherapy schedule, the presence of an early acute response and genetic background, to the risk of specific late injury phenotypes.

## MATERIALS AND METHODS

### Patients and methods

The study has been undertaken with the participation of patients attending the oncology departments of Leicester Royal Infirmary, Glenfield Hospital, Leicester and Nottingham City Hospital, UK. Full ethical and local trust approval was obtained from the relevant ethics committees and trust Research and Development departments. All patients gave written informed consent before entry into the study, and underwent an examination of the affected area and the recognised features of late radiation effects scored by the SOMA and/or RTOG scales. Seven patients refused to take part in the study.

Tumour-free breast cancer patients were recruited sequentially in the follow-up clinic of one consultant in Leicester, UK (IP). The majority (155/167) were treated more than 4 years previously (median=6.1 years), but 12 additional patients who were known to have late effects were examined at the City hospital, Nottingham (*n*=5), and the Leicester Royal Infirmary (*n*=7), UK between 2.3 and 3.6 years after treatment. All patients were asked about potential radiosensitising comorbid diseases such as diabetes or collagen vascular disorders and a family history of abnormal reactions to radiotherapy. Initial surgery was either a lumpectomy (*n*=116) or mastectomy plus axillary dissection (*n*=47). Two patients had a lumpectomy but then went on to have a mastectomy, and two had no surgery.

Radiotherapy (6 MV X-rays) was given either after mastectomy or lumpectomy using tangential fields, and all patients received similar field sizes. All patients also had a 1.5 cm thick strip of wax 4 cm in width applied to either the lumpectomy or mastectomy scar to reduce ‘skin sparing’ during every second fraction. A total of 119 patients received the then standard departmental protocol of 45 Gy in 20 fractions in 4 weeks given to either the breast or chest wall. Seventy-one of these patients who had been treated by local excision received a boost of 15 Gy in five fractions in 1 week using 8–12 MeV electrons to the site of the lumpectomy. Boost sizes were between 6 × 6 and 8 × 10 cm. Ten patients received 40 Gy in 15 fractions as part of the START B trial (NCRN Trial Standardisation of Breast Radiotherapy, 1998). Thirty-three patients were treated with a whole breast dose of 50 Gy in 25 fractions either as part of the START trial or following the publication of the European Organisation of Cancer Radiotherapy and Breast Cancer Groups study (EORTC) (Bartelink *et al*, 2001), which identified a group of patients (node negative, complete excision aged <50) who did not require a local boost (Table 1). The mean age at treatment was 56.92 (range 30–78).

The majority of patients (157/167) were Caucasians, but nine patients were of Asian descent and one patient of African Caribbean descent. Twenty-seven patients received anthracyline-based chemotherapy (two of which also received CMF), and 18 patients received non-anthracyline-based chemotherapy before radiotherapy, and 151 patients were further treated with tamoxifen (one stopped during treatment).

Twenty-four patients were positive for an acute reaction within 90 days of treatment, and three of these did not complete the radiotherapy schedule. However, including the 15 Gy boost, only one of the three acute responders received significantly less (16.73 Gy) than a total 2 Gy equivalent dose of 40 Gy.

#### Sample collection and preparation

DNA was collected from the patients for genetic analysis by means of a buccal swab. DNA was extracted from the buccal samples using the QIAamp DNA mini kit (QIAGEN GmbH, Germany), using the standard manufacturers’ protocol. DNA was PCR amplified and analysed for QTG restriction fragment length polymorphisms using published PCR primers and PCR conditions, and restriction enzymes: *TGFβ1* (C-509T) (Quarmby *et al*, 2003); X-ray repair cross-complementing 1 (*XRCC1*; R399Q) and apurinic/apyrimidinic endonuclease (*APE-1*; D126E) (Hu *et al*, 2001); dihydrofolate reductase (*DHFR*; 15 bp intron 1 deletion) (Johnson *et al*, 2004); the *CX3CR1* (G745A) fractalkine receptor (McDermott *et al*, 2001); epoxide hydrolase (*Hyl-1*; Y113H) (Lebailly *et al*, 2002); and methionine synthetase (*MS*; A2756G) and methylenetetrahydrofolate reductase (*MTHFR*; C667T) (Linnebank *et al*, 2004).

#### Statistical analysis

The *G*-test was used to examine the relationship between genotype and phenotype. The *G*-tests are maximum likelihood statistical significance tests (log-likelihood ratio) using the William's correction for small sample size (Sokal and Rohlf, 1995). Allele frequencies were examined by calculating the odds ratios (OR), with 95% confidence intervals (95% CI), for the frequency of the polymorphic allele in one group relative to another.

## RESULTS

A total of 167 breast cancer patients that were treated with radiotherapy were recruited and examined for late normal tissue damage. The objective SOMA scales (0–4) (Pavy *et al*, 1995) were used to score for fibrosis, atrophy and telangiectasia severity during a detailed physical examination and represent separate phenotypic measures of late normal tissue damage. The SOMA scores of 1 represent subtle examiner-dependent changes that are inconclusive and in this study are either excluded or included in the group of patients that show no signs (SOMA 0) of a particular injury phenotype. In contrast, the SOMA scores of 2–4 represent unambiguous evidence of late tissue injury. Patients were also scored using the comparatively nonspecific RTOG scales, but were non-informative in all the association studies performed (data not shown).

Factors that might influence the risk and severity of late normal tissue damage include anatomical differences between individuals, the initial surgery, treatment regimes (whether or not the patient received chemotherapy or an electron boost), whether the patient had an early acute reaction and whether the individual patient was genetically predisposed. Chemotherapy may increase late effects (Fiets *et al*, 2003; Shanley *et al*, 2006) especially anthracycline containing regimens but the number of patients that received anthracyline and non-anthracyline-based chemotherapy in this study is too small for any meaningful statistical analysis to compare the two chemotherapies (Table 1).

### Radiation dose

The majority (165/167) of patients received a very similar total 2 Gy dose fraction equivalent (45–50 Gy; Table 1), so dose differences are largely due to whether the patient received an additional 15 Gy electron boost (18/2 Gy equivalent). The area irradiated in all patients was very similar (6 × 6–8 × 10 cm), and the late injury phenotypes readily scored within that area by sight and/or touch.

Excluding the one patient who received significantly less than 45 Gy because of a severe acute reaction to radiotherapy (dose 15.75 Gy; Table 1), patients who received a 15 Gy boost (*n*=75) were compared with patients who did not receive a boost (*n*=91) (Table 2). Patients that manifested no signs of late injury (SOMA score 0) were compared with patients that clearly showed signs of late tissue injury (SOMA score 2–3). No association between fibrosis and the 15 Gy boost was observed (*P*=0.47) and a similar result was obtained for atrophy (*P*=0.28; data not shown). In contrast, the 15 Gy boost was weakly associated with an increased risk of telangiectasia (OR 2.4; 95% CI 1.1–5.4; *P*=0.033), consistent with clinical evidence that telangiectasia is usually confined to the boost-exposed region of the breast.

### Late injury severity and early acute reaction

Twenty-five patients had an unusually severe acute reaction (erythema, moist desquamation) within 90 days of treatment, so the presence or absence of this early radiation phenotype was compared with the late objective SOMA scores (Table 3). Comparing unaffected patients (SOMA score 0) and clearly affected patients (SOMA score 2–4) indicates that an early acute reaction increases the risk of long-term fibrosis by an OR of 8.5 (95% CI 2.64–28.5; *P*=0.00004), and increases the risk of long-term atrophy by 4.7 (95% CI 1.25–17.64; *P*=0.02). Telangiectasia as a late normal tissue injury phenotype was not associated with an early acute reaction.

In conclusion, within the 45–50 Gy dose range (20–25 fractions) used to treat breast cancer patients in this study, an additional 15 Gy boost appears to contribute to an increased risk of telangiectasia. Conversely, a boost does not predispose to fibrosis (or atrophy) but an acute reaction does. These findings, which are based on relatively small numbers of patients, are in disagreement with earlier studies of post-mastectomy patients (Bentzen and Overgaard, 1991; Bentzen *et al*, 1993) and thus need to be independently confirmed.

### Genetic association between genotype and phenotype

Patients were genotyped for eight functional SNPs in candidate genes. All polymorphisms were assessed by PCR amplification of buccal DNA and analysed using established Restriction Fragment Length Polymorphisms (RFLP). Transforming growth factor-*β*1 polymorphisms were confirmed by direct sequencing.

The frequencies of the variant alleles were comparable with those reported in other studies (Hu *et al*, 2001; McDermott *et al*, 2001; Lebailly *et al*, 2002; Quarmby *et al*, 2003; Johnson *et al*, 2004; Linnebank *et al*, 2004), and all SNPs were in Hardy–Weinberg equilibrium (Table 4).

The genes and corresponding SNP were chosen because *XRCC1* (R399Q) and *APE-1* (D126E) have been implicated in DNA repair (Hu *et al*, 2001; Chang-Claude *et al*, 2005; De Ruyck *et al*, 2005), *TGFβ1* (C-509T) and *CX3CR1* (G745A) have been implicated in inflammation and fibrosis (Zugmaier *et al*, 1991; Randall and Coggle, 1995; Anscher *et al*, 1997; Li *et al*, 1999; Martin *et al*, 2000; McDermott *et al*, 2001; Gauldie *et al*, 2003), *MTHFR* (C667T), MS (A2756G) and *DHFR* (19 bp del intron 1) involved in folate metabolism (Johnson *et al*, 2004; Linnebank *et al*, 2004), epoxide hydrolase (*Hyl1* Y113H) implicated in oxidative stress (Lebailly *et al*, 2002), and *XRCC1* (R399Q) have been associated with susceptibility to breast cancer (Moullan *et al*, 2003). Additionally, several gene polymorphism have previously been implicated in late radiation-induced tissue damage (Awad *et al*, 1988; El-Gamel *et al*, 1999; Grainger *et al*, 1999; Quarmby *et al*, 2003; Andreassen *et al*, 2005; De Ruyck *et al*, 2005, 2006; Brem *et al*, 2006), and *TGFβ1* and *Hyl1* are induced following exposure to ionising radiation (Anscher *et al*, 1997; Martin *et al*, 1997).

Out of the eight SNP examined, only the *TGFβ1* (C-509T) and *XRCC1* (R399Q) polymorphisms showed any significant association with late tissue injury in this study.

### Fibrosis and telangiectasia

Subjective Objective Management Analytical scores for fibrosis and telangiectasia were analysed by comparing clearly affected (SOMA score 2–4) and unaffected patients (SOMA score 0 or 0–1). As shown in Table 5, the *TGFβ1* (C-509T) polymorphism was significantly associated with an increased risk of fibrosis, whereas the *XRCC1* (R399Q) polymorphism was significantly associated with an increased risk of telangiectasia. No significant genetic association was observed between atrophy and genotype (data not shown).

As the 15 Gy boost also appears to increase the risk of telangiectasia (Table 2), the XRCC1 (R399Q) association was performed excluding those patients who had received a boost (*n*=75) to determine whether the two risk factors were distinct. Similarly, an acute reaction to radiotherapy and the TGF*β*1 C-509T polymorphism increase the risk of fibrosis (Table 3), so patients who manifested an acute response were excluded to separate the two potentially distinct risk factors.

As shown in Table 6, there is a statistically significant excess of XRCC1 R399Q RQ heterozygotes in patients with telangiectasia (*P*=0.01; *G*-test), and an excess of TT homozygotes in patients with fibrosis (*P*=0.003; *G*-test), suggesting that the risk of telangiectasia and fibrosis as late normal tissue injury phenotypes is determined by multiple independent factors.

A boost and the *XRCC1* (R399Q) polymorphism each contribute to the risk of telangiectasia, whereas an acute reaction and the *TGFβ1*(C-509T) polymorphism make independent contributions to the overall risk of fibrosis. Significantly, although the risk of fibrosis and atrophy are both associated with an early acute response to radiotherapy (Table 3), atrophy was not associated with either the *XRCC1* (R399Q) or *TGFβ1* (C-509T) polymorphisms, evidence that additional distinct genetic risk factors underlie the risk of atrophy as a late radiation injury phenotype.

The reduced sample size reduces the statistical power of any association, so the results are at best suggestive and require independent confirmation.

### Combined *TGFβ1* (C-509T) analyses

Quarmby *et al* (2003), first reported a significant association between the *TGFβ1* (C-509T) polymorphism in Caucasian breast cancer patients who had received a similar radiotherapy dose (40 Gy in 15 fractions) to those used in this study (Table 1). Fibrosis was also scored using the SOMA scales, so the two studies are very similar. Quarmby *et al* (2003) compared patients with fibrosis (*n*=14; SOMA 2–4) to patients without fibrosis (*n*=87; SOMA 0), and our data for the SOMA fibrosis scores can also be divided into those that clearly show no fibrosis (score 0; *n*=115) and those that unambiguously show signs of fibrosis (scores 2–4; *n*=20). The data for the two studies were therefore combined to significantly increase the number of patients analysed (*n*=236; Table 7).

The combined data (Tables 7 and 8) reveal a highly significant association (*P*=0.00006) with an OR of 3.06 (95% CI: 1.74–5.3) for patients carrying at least one variant allele (CT or TT) compared with the homozygous wild-type CC genotype, and therefore this increased risk applies to ∼56% (132/236) of breast cancer patients. Furthermore, the 8% of patients that are homozygous for the variant allele (TT) carry a much higher risk of fibrosis than CC homozygotes (OR 14.7; 95% CI 3.8–60.3) and a lesser but still significant increased risk compared with CT heterozygotes (OR 4.45; 95% CI 1.4–14). Together with the observation that CT heterozygotes have a 3.3-fold increased risk of fibrosis than CC homozygotes, it suggests that the increased risk with each variant allele is additive.

## DISCUSSION

Late normal tissue injury in breast cancer patients that have received standard radiotherapy treatment (40–50 Gy) using iso-effective fractionated schedules, with or without an additional 15 Gy boost, is a combination of phenotypes with distinct underlying causes. For example, the risk of telangiectasia appears to increase if the patient received a 15 Gy boost and this has been self-evident in the clinic as the telangiectasia is usually confined to the area of the breast that received the boost. However, there are also dose-independent genetic risk factors as evidenced by the association of the *XRCC1* (R399Q) DNA repair gene polymorphism with increased risk of telangiectasia irrespective of the patient having received a boost. Similarly, the risk of fibrosis appears to increase if an early acute radiation reaction is observed with 90 days of therapy, but the *TGFβ1* (C-509T) variant allele is another major independent genetic contributor to the risk of fibrosis as a late radiation injury phenotype. Atrophy is also associated with an early acute radiation response, but as atrophy is not associated with any of the QTG analysed, including *XRCC1* (R399Q) or *TGFβ1* (C-509T), the identity of the variant gene(s) that predispose patients to atrophy as a distinct radiation injury phenotype is unknown. Similarly, the contribution of different chemotherapeutic regimes to risk of late tissue injury could not be addressed in this study.

Breast cancer is the most common female cancer in the UK and the prevalence of radiotherapy treatment in this disease is high. In 2000, there were 40 470 new cases of this disease in women and it is estimated that the lifetime risk of women in the UK developing breast cancer is 1 : 9 (CRUK, 2004). Radiotherapy substantially reduces the risk of local recurrence after surgery with a more modest reduction in cancer mortality offset by an increase in contralateral breast cancer and cardiac disease, which is more marked in older trials (EBCTCG, 2005). Virtually, all patients treated by breast conservation and patients with high risk factors for local recurrence after mastectomy receive radiotherapy. A significant proportion (44% in this study) of patients nevertheless remains at risk of recurrence and therefore receive an additional 15 Gy electron boost. Although these therapy schedules have significantly increased long-term survival, normal tissue radiation injury lasts a lifetime and is therefore increasingly a factor that must be considered in treatment choice as it can affect quality of life.

Telangiectasia in most cases is only unsightly. However, fibrosis is often associated with painful tender breasts as evidenced by the significant association of fibrosis and pain in this group of patients (*P*=0.003; data not shown). It is noteworthy that although the severity of pain rarely exceeds discomfort ameliorated by simple analgesia, these symptoms are likely to be life-long and affect quality of life. It is possible that some patients with a high-risk genotype that predisposes them to late radiation effects could avoid radiotherapy. In other cases, the advantages of radiotherapy may outweigh the risks of fibrosis and discomfort and such patients may need to be warned of the benefits and additional risks associated with radiotherapy.

Quarmby *et al* (2003) originally reported an association of the *TGFβ1* (C-509T) gene promoter polymorphism and increased risk of fibrosis as a late radiotherapy injury phenotype. We have combined the data in both studies (Tables 7 and 8) to more than double both the total number of patients analysed (*n*=236) and the number of patients with fibrosis (*n*=38). This combined analysis revealed a highly significant three-fold increased risk of fibrosis following radiotherapy if a breast cancer patient carries at least one variant *TGFβ1* (−509T) allele (*P*=0.00006) and this applies to over 50% of breast cancer patients. Approximately 8% of breast cancer patients are homozygous (TT) for the variant allele and they have a 15 fold increased risk of fibrosis (*P*=0.000003) compared to patients that are homozygous for the wild-type allele (CC). Together with any evidence of an early acute response to radiotherapy, a patient's *TGFβ1* (−509) genotype might be useful in predicting the risk of fibrosis in the long term in a significant number of patients.

One recent study could not replicate the *TGFβ1* (−509) SNP association with fibrosis in a retrospective analysis of breast cancer patients (Andreassen *et al*, 2003, 2005, 2006). The reason is unclear although differences in the two Danish studies (and the UK studies; Table 7) include the fact that all patients in the latter Danish 2006, study had mastectomies, were specifically selected retrospectively because they exhibited fibrosis, and were treated with higher radiation doses per fraction than the original 2005 study, which used changes in breast appearance as the phenotype. Thirteen percent of Danish breast cancer patients in the 2006 study carried the TT genotype (Andreassen *et al*, 2006), which is higher than in the UK breast cancer patients as a whole (7–9%, Table 7), but lower than the 26% of the UK patients that were positive for both fibrosis and the TT genotype (Table 7). Thus, the higher fraction doses used in a significant proportion of the Danish patients in the 2006 report may be masking any genetic contribution. Furthermore, as the Danish patients were specifically selected in a retrospective study (2006), because they exhibited fibrosis as a late radiation injury, their genotype distribution cannot be combined with the UK studies (Table 7) where only a very small proportion of patients were selected and the majority of patients exhibited no late effects (Tables 2, 3, 4, 5 and 6) (Quarmby *et al*, 2003). The precise patient genotype frequencies are not given in the Danish 2005 publication (Andreassen *et al*, 2005), so the data as published cannot be compared or combined with the UK studies. Nevertheless, if the patient genotypes were available from the 2005 study, which compares affected (*n*=26) and unaffected (*n*=26) patients who received 39–50 Gy total dose, they could be pooled with the UK data, as the experimental designs are so similar.

The observed contributions to fibrosis risk of an early severe acute reaction, which can trigger an inflammatory response, and the *TGFβ1* (C-509T) SNP, which has been associated with increased serum levels of the pro-inflammatory TGF*β*1 cytokine following radiotherapy, are consistent with the established link between fibrosis and inflammation (Awad *et al*, 1988; Zugmaier *et al*, 1991; Anscher *et al*, 1997; Martin *et al*, 1997, 2000; Grainger *et al*, 1999; Li *et al*, 1999). Additional genetic factors are presumably responsible for the radiosensitivity that causes the severe acute reaction and a number of variant genes have been implicated in abnormal cell radiosensitivity (Moullan *et al*, 2003; Chang-Claude *et al*, 2005; De Ruyck *et al*, 2005; Fernet and Hall, 2005), but none of the eight candidate gene SNP analysed in this study was significantly associated with an early acute reaction. The observed increased risk of telangiectasia in patients, who received an additional 15 Gy boost and/or are deficient in DNA repair owing to the inheritance of a variant *XRCC1* (R399Q) allele, is consistent with the association of telangiectasia with vascular endothelial cell damage (Quarmby *et al*, 1999).

Multiple testing has been performed on the same patient samples so false positive or negative results are likely. As the association between fibrosis and the *TGFβ1* (−509) polymorphism in breast cancer patients is a confirmation of previous independent analyses (Quarmby *et al*, 2003; Andreassen *et al*, 2005), the Boniferroni correction factor does not apply in this case, but the association would still be significant (*P*<0.05) if the correction factor was applied, as it would be for the association between *XRCC1* (R399Q) and telangiectasia. Nevertheless, the number of patients assessed is relatively small, so further analyses using an independent cohort of breast cancer patients are required to confirm the apparent association between an early acute reaction and late fibrosis/atrophy, and between the additional 15 Gy boost, *XRCC1* (R399Q) and telangiectasia.

## Figures and Tables

**Table 1:** Radiotherapy and chemotherapy

			**No. of patients**		**15 Gy Electron boost no. of patients**	
**Total dose (Gy)**	**No. of fractions**	**2 Gy equivalent dose (*α*/*β* 2)**	**Chemotherapy**	**Tamoxifen**	**No boost**	**Boost**
15.75^a^	7	16.73	0	1	1	0
34^a^	17	34	0	0	0	1
38^a^	17	40.64	0	1	0	1
40	15	46.6	2	10	9	1
45	20	47.81	33	109	48	71
48	24	48	1	1	1	0
50	25	50	9	29	33	1

a Patients who did not complete the planned radiotherapy schedule due to a severe early reaction to radiotherapy.

**Table 2:** Risk of injury phenotypes and the 15 Gy electron boost

	**Fibrosis number of patients**		**Telangiectasia number of patients**	
**SOMA score**	**No boost**	**Boost**	**No boost**	**Boost**
0	67	47	66	44
1	14	18	10	7
2	8	8	11	14
3	2	2	4	10

**Table 3:** Risk of late injury phenotypes and an early acute reaction

	**Fibrosis (patients)**		**Telangiectasia (patients)**		**Atrophy (patients)**	
**SOMA score**	**Not acute**	**Acute**	**Not acute**	**Acute**	**Not acute**	**Acute**
0	103	12	97	14	99	14
1	29	3	12	5	34	5
2	8	8	22	3	8	4
3	2	2	11	3	1	2

**Table 4:** Variant allele frequencies in the breast cancer patients

**SNP**	**Variant allele frequency**	**Hardy–Weinberg (*P*)**
*TGFβ*-*1* C-509T	0.33	0.22
*XRCC1* R399Q	0.37	0.51
*APE-1* D126E	0.34	0.39
*DHFR* (del)	0.46	0.35
*CX3CR1* G745A	0.28	0.88
*Hyl-1* Y113H	0.33	0.25
*MS* A2756G	0.23	0.09
*MTHFR* C667T	0.33	0.61

**Table 5:** Genetic association of the SOMA fibrosis and telangiectasia scores

	***G*-test. Fibrosis**		
	***P***	**Scores compared**	**Patient number**
*TGFβ1* C509T	0.0041	0 *vs* 2–4	115 *vs* 20
	0.0032	0–1 *vs* 2–4	147 *vs* 20
			
	***G*-test. Telangiectasia**		
	***P***	**Scores compared**	**Patient number**
*XRCC1* 399	0.01	0 *vs* 2–4	111 *vs* 39
	0.006	0–1 *vs* 2–4	128 *vs* 39

**Table 6:** Genotype distributions for fibrosis and telangiectasia excluding patients that received a boost, or manifested an early acute reaction

	**Telangiectasia: patients who did not receive a boost *XRCC1* (R399Q) genotype**			**Fibrosis: patients who did not show an acute reaction *TGFβ1* (C-509T) genotype**		
**Soma score**	**RR**	**RQ**	**QQ**	**CC**	**CT**	**TT**
0	33	25	9	49	47	7
2–4	2	9	4	0	7	3

**Table 7:** Combined analysis of the *TGFβ1* (C-509T) polymorphism and fibrosis

	**Quarmby *et al*, 2003 (patients)**		**This study – Table 4 (patients)**		**Combined (patients)**	
**C-509T genotype**	**No fibrosis**	**Fibrosis**	**No fibrosis**	**Fibrosis**	**No fibrosis**	**Fibrosis**
CC	45	4	53	2	98	6
CT	40	5	54	14	94	19
TT	2	5	8	4	10	9

**Table 8:** Risk of fibrosis according to *TGFβ1* genotype

	**Odds ratio**	**95% CI^a^**	***P***
CC *vs* (CT+TT)	3.06	1.7–5.3	0.00006
(CC+CT) *vs* TT	7.27	2.4–21.8	0.00005
CC *vs* TT	14.7	3.8–60.3	0.000003
CT *vs* TT	4.45	1.4–14.0	0.007
CC *vs* CT	3.3	1.2–9.7	0.002

a 95% confidence intervals.
